# Association of Dietary Cholesterol Intake With Risk of Gastric Cancer: A Systematic Review and Meta-Analysis of Observational Studies

**DOI:** 10.3389/fnut.2021.722450

**Published:** 2021-08-12

**Authors:** Peng Miao, Lin Guan

**Affiliations:** ^1^General Surgery Department, The First Hospital of China Medical University, Shenyang, China; ^2^Gastroenterology Department, The First Hospital of China Medical University, Shenyang, China

**Keywords:** dietary cholesterol, gastric cancer, meta-analysis, dose-response, systematic review, diet

## Abstract

**Background:** Many case–control studies have investigated the association between dietary cholesterol and gastric cancer, yielding inconsistent findings. We carried out a systematic review and meta-analysis of observational studies to assess the relationship between dietary cholesterol intake and gastric cancer among adults.

**Methods:** PubMed, Scopus, and Google Scholar were systematically searched to identify articles that evaluated the association of dietary cholesterol with gastric cancer up to May 2021. Pooled odds ratio (ORs) and 95% confidence intervals (CIs) were computed using random-effects models. Dose–response analysis was used to explore the shape and strength of the association.

**Results:** Fourteen case–control studies with 6,490 gastric cancer patients and 17,793 controls met our inclusion criteria. In the meta-analysis of the highest vs. the lowest dietary cholesterol categories, a significantly higher (~35%) risk of gastric cancer was observed in association with high cholesterol consumption (pooled OR: 1.35, 95% CI: 1.29–1.62, *I*^2^ = 68%; 95%CI: 45–81%). Subgroup analysis also showed this positive relationship in population-based case–control studies, those conducted on non-US countries, those with a higher number of cases and high-quality studies, those that collected dietary data via interviews, studies not adjusted for *Helicobacter pylori* infection, and studies where the body mass index was controlled. Besides, a non-linear dose–response association was also identified (*P* = 0.03).

**Conclusion:** This study demonstrated that dietary cholesterol intake could significantly augment the risk of gastric cancer in case–control studies. Prospective cohort studies with large sample sizes and long durations of follow-up are required to verify our results.

## Introduction

Gastric cancer (GC) represents the fifth most common cancer and the third leading cause of cancer deaths in males and females worldwide, with nearly one million new cases and 723,100 deaths from GC every year (1). Given the increasing prevalence of GC and its mortality, new strategies are necessary to minimize the disease burden. *Helicobacter pylori* infection, high alcohol consumption, obesity, smoking, and dietary factors are the main risk factors of GC (2, 3). Numerous studies have shown the association between nutritional factors and GC (3, 4). In fact, one meta-analysis found that the total dietary fat was positively associated with GC (5).

Cholesterol is a common nutrient in the human diet, with eggs, red meat, dairy products, fish, and poultry representing its major sources (6). It has been indicated that dietary cholesterol can increase serum cholesterol, low-density lipoprotein (LDL), and high-density lipoprotein (HDL) cholesterol concentrations (7). Hypercholesterolemia may be involved in cancer development via a rise in the level of inflammatory markers (8).

Some meta-analyses demonstrated that high dietary cholesterol intake increases the risk of ovarian, breast, pancreatic, and esophageal cancers (9–12). However, the association between dietary cholesterol intake and GC risk remains controversial. Some case–control studies have indicated a positive relationship (13, 14), while others showed no association (15, 16). Based on our knowledge, there is no systematic review and meta-analysis to summarize the findings regarding dietary cholesterol intake and GC.

Therefore, considering the conflicting results and increasing incidence of GC worldwide, we carried out a systematic review and meta-analysis to provide a quantitative synthesis of the existing data on the association between dietary cholesterol intake and the risk of GC in adults. Furthermore, we aimed to assess the shape and strength of the dose–response association between dietary cholesterol intake and GC.

## Methods

The framework of this review was structured according to the Preferred Reporting Items for Systematic Review and Meta-Analysis (PRISMA) statement [(17); Supplementary Table 1].

### Search Strategy

An advanced systematic search of PubMed, Scopus, and Google Scholar was performed without any restrictions (including language) using Medical Subject Heading (MeSH) and related keywords to discover relevant articles published until May 2021. The search terms were:[(“cholesterol^*^” OR “dietary cholesterol” OR “cholesterol intake” OR “cholesterol consumption” OR “fat intake” OR “dietary fat”) AND (“gastrointestinal cancer” OR “gastrointestinal carcinoma” OR “gastrointestinal neoplasm” OR “gastrointestinal adenocarcinoma” OR “gastrointestinal tumor” OR “gastric cancer” OR “gastric carcinoma” OR “gastric neoplasm” OR “gastric adenocarcinoma” OR “gastric tumor” OR “stomach cancer” OR “stomach carcinoma” OR “stomach neoplasm” OR “stomach adenocarcinoma” OR “stomach tumor”)]. Besides, the reference lists of the relevant articles and reviews were manually inspected in order to complete the search. The protocol of this investigation was registered in the International Prospective Register of Systematic Reviews (PROSPERO) (CRD42021255008).

### Inclusion Criteria

Studies with the following criteria were included: (1) a prospective cohort or case–control design; (2) participants were aged ≥18 years; (3) provided risk estimates, including relative risk (RR), hazard ratios (HRs), and odds ratios (ORs) with 95% confidence intervals (CIs) to evaluate the association between dietary cholesterol intake and GC. When several studies used one dataset, we selected the one with the greatest number of cases. Two independent authors reviewed articles according to the mentioned items. If they encountered any controversy, the principal investigator resolved the issue.

### Exclusion Criteria

Unpublished papers, abstracts, ecological studies, reviews, letters, and comments were excluded. Furthermore, studies that considered another cancer along with GC and articles that used population-attributable risks to assess the association were removed.

### Data Extraction

The following items were extracted from each included study: name of the first author, publication year, study location, study design, gender, age (mean/range), the total number of participants, cases, controls, median/range of cholesterol intake in each category, most adjusted RRs, HRs, or ORs and 95% CIs, dietary assessment method, outcome assessment approach, and adjustments. Two authors extracted the data independently, and the corresponding author resolved any disagreements.

### Risk of Bias Assessment

The risk of bias for each study was determined using the Newcastle–Ottawa scale (18). Each study received an overall score between 0 and 9 according to the selection of case and control groups, comparability, and ascertainment of exposure and outcome. A total score of ≥7 was representative of a high-quality study.

### Statistical Methods

We used a random-effects model to compute summary risk estimates and 95% CIs for the associations between dietary cholesterol intake (highest vs. lowest categories) and GC. Between-study heterogeneity was assessed using the *I*^2^ index and its CI (19). In terms of between-study heterogeneity, *I*^2^-values of 25–50%, 50–75%, and >75% were considered as low, moderate, and high heterogeneity, respectively (20). To discover potential sources of heterogeneity, subgroup and meta-regression analyses were conducted based on study design (population-based case–control studies, hospital-based case–control studies), number of cases, study quality, exposure reporting method, and adjustments (yes/no) for *H. pylori* infection, energy intake, and body mass index (BMI). In studies that reported the separate risk estimates for each gender, we first combined the risk estimates using a fixed model and then entered them into the final analysis.

We used the generalized least-squares trend estimation method to conduct a linear dose–response analysis (21, 22). Estimated study-specific slope lines were combined to create an average slope using a random-effects model. Studies that reported the number of cases and controls, the mean/median intake of cholesterol, and the RRs with a 95% CI for at least three exposure categories were eligible for dose–response analysis. For studies that only reported the total number of cases and controls, we estimated the number of cases and controls in each category by dividing the total number by the number of categories.

In non-linear dose–response analysis, exposures were modeled using restricted cubic splines with three knots at percentiles of 10, 50, and 90% of the distribution. The correlation within each set of provided risk estimates was taken into account, and the study-specific estimates were combined using a one-stage linear mixed-effects meta-analysis. The significance for non-linearity was determined by null hypothesis testing, where the coefficient of the second spline was considered equal to zero.

Publication bias was identified using Egger's linear regression test and funnel plot inspection (23). Sensitivity analysis was done using a random-effects model to assess the impact of each study on the overall risk estimate. This analysis was carried out by excluding each study and reanalyzing the data. All analyses were done using STATA version 16.0, and *P* < 0.05 was considered statistically significant for all tests.

## Results

**Figure 1:**
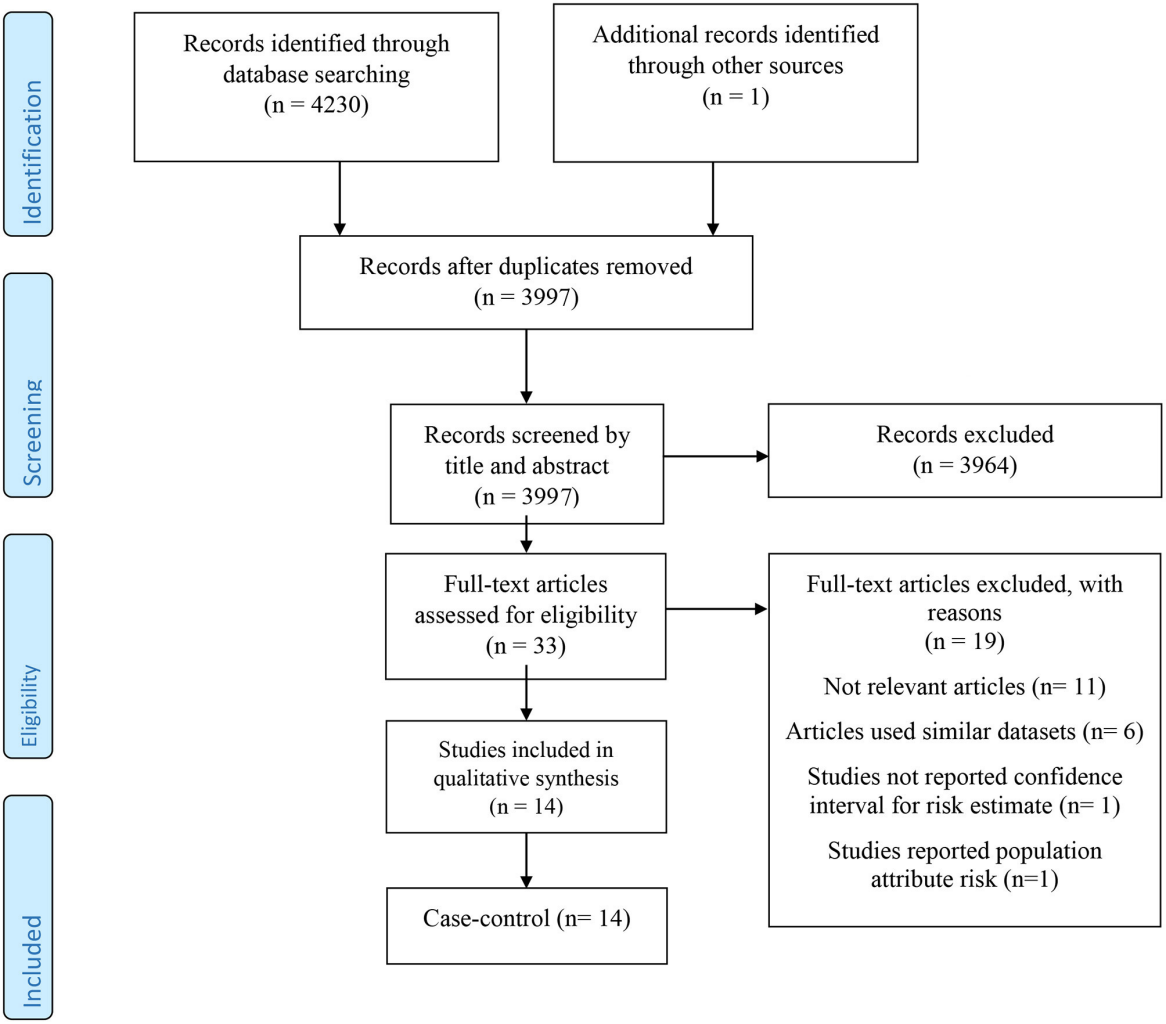
The flow diagram of study selection.

After removing 234 duplicate articles from a total of 4,231 papers identified through the initial search, 3,997 papers remained for reviewing the title and abstract. At this stage, 3,964 publications were excluded, and the full texts of 33 remaining articles were checked. Among 19 studies that were eliminated in this step, six used similar datasets, one did not report the CI, 11 were irrelevant, and one reported the population-attributable risk. Finally, 14 case–control studies were eligible for our systematic review and meta-analysis [(13–16, 24–33); Figure 1].

Study-specific characteristics are illustrated in Table 1. Nine population-based case–control (13, 14, 16, 24, 26, 29–31, 33) and five hospital-based case–control studies (15, 25, 27, 28, 32) published from 1990 to 2020 met our criteria. In total, 6,490 GC patients and 17,793 controls aged between 20 and 98 years were included. Studies were conducted in United States (*n* = 4) (13, 25, 31, 33), Italy (*n* = 2) (15, 24), China (*n* = 2) (14, 30), Canada (*n* = 1) (26), Mexico (*n* = 1) (29), Poland (*n* = 1) (16), South Korea (*n* = 1) (27), Serbia (*n* = 1) (28), and Iran (*n* = 1) (32). All studies were conducted on both genders and used food frequency questionnaires (FFQs) for dietary assessment. In 11 case–control studies, matching for age and gender was carried out between the case and control groups (13–16, 24, 26–30, 33). Furthermore, some important covariates, including *H. pylori* infection (*n* = 4) (14, 27, 31, 33), total energy intake (*n* = 13) (13–16, 24–26, 28–33), BMI (*n* = 8) (13–15, 24–26, 31, 32), alcohol consumption (*n* = 6) (13, 14, 25, 26, 30, 31), and smoking (*n* = 12) (13–16, 25, 26, 28–33), were adjusted in the analysis. According to quality assessment findings, nine studies were classified as high-quality (score of ≥7) studies [(13, 15, 16, 24, 26, 29, 30, 32, 33); Supplementary Table 2].

**Table 1:** Characteristics of included studies on the association between cholesterol intake and gastric cancer in adults aged >18 years in case-control studies.

**References**	**Country**	Age^*^	**Age (cases)**	**Age (controls)**	**Cases *n***	**Control *n***	**Exposure assessment**	**Median/cutoff point**	**OR (95%CI)**	**Adjustment**
Buiatti et al. (24)	Italy	<70	NR	NR	M/F:1,016	M/F:1,159	FFQ/interview	142 mg/d 199 mg/d 242 mg/d 300 mg/d 434 mg/d	1 0.9 (0.7–1.2) (0.8–1.4) 1.3 (0.9–1.7) 1.2 (0.8–1.6)	Age, sex, area, place of residence, migration from south, socioeconomic status, familial GC history, and BMI
Hu et al. (26)	Canada	20–76	61.9	56.8	M:802 F:379	M:2,547 F:2,492	FFQ/self-report	≤ 966.26 mg/wk 966.26–1412.75 mg/wk 1412.75–1880.26 mg/wk ≥1880.26 mg/wk	1 1.10 (0.87–1.39) 1.41 (1.10–1.80) 1.60 (1.21–2.13)	Sex, age group, province, education, body mass index, alcohol drinking, pack year smoking, total of vegetable and fruit intake, saturated fat, and total energy intake
Kim et al. (27)	South Korea	57.2 ± 0.84	57.2 ± 1.19	57.2 ± 1.20	M:92 F:44	M/F:136	M:92 F:44	Cases: 123 mg/d 174 mg/d 240 mg/d Controls: 140 mg/d 185 mg/d 248 mg/d	1 0.62 (0.34–1.13) 0.51 (0.25–1.05)	Age, sex, socioeconomic status, family history, refrigerator use, and *Helicobacter pylori* infection.
Lazarevic et al. (28)	Serbia	NR	NR	NR	M/F:102	M/F:204	FFQ/interview	NR	1 0.92 (0.86–3.59) 0.79 (0.37–2.28)	Age, sex, residence, education, physical activity, total energy intake, tobacco smoking, and history of cancer in the first degree
Lissowska et al. (16)	Poland	NR	NR	NR	M:175 F:99	M:304 F:159	FFQ/interview	<144.6 mg/d 144.6–167.9 mg/d 168–196.1 mg/d >196.1 mg/d	1 1.08 (0.71–1.64) 0.94 (0.61–1.43) 1.57 (0.89–2.78)	Age, sex, education, smoking, and calories from foods
López-Carrillo et al. (29)	Mexico	>20	24–88	20–98	M:121 F:99	M:301 F:451	FFQ/interview	≤ 190.5 mg/d 190.51–264.03 mg/d 264.04–359.51 mg/d ≥359.52	1 1.58 (0.87–2.87) 1.77 (0.96–3.24) 2.39 (1.23–4.64)	Age, gender, total calories, chili-pepper consumption, socio-economic status, cigarette smoking, salt consumption, history of peptic ulcer, type of interview, duration of interview, place of interview
Lucenteforte et al. (15)	Italy	22–80	22–80	22–80	M:143 F:87	M:286 F:261	FFQ/interview	NR Per 105 mg/d	1 0.97 (0.64–1.47) 1.27 (0.86–1.89) Continuous: 1.11 (0.94–1.32)	Age and sex, year of interview, education, physical activity, body mass index, tobacco smoking, family history of stomach cancer and total energy intake
Mayne et al. (13)	US	30–79	64.2	61.8	M:467 F:140	M:543 F:145	FFQ/interview	NR	Gastric cardia adenocarcinoma 1 1.50 (1.19–1.90) Non-cardia gastric cancer 1 1.68 (1.35–2.09)	Sex; site; age; race; proxy status; income; education; usual body mass index; cigarettes/day; years of consuming beer, wine, and liquor; and energy intake.
Qiu et al. (30)	China	NR	30–85	28–82	M:81 F:22	M:95 F:38	FFQ/interview	NR	Males: 1.0 1.08 (0.40–2.87) 2.53 (0.99–6.44) 2.76 (1.01–7.53) Females: 1.0 6.05 (0.53–69.17) 5.31 (0.44–63.44) 11.9 (0.97–146.53)	Age, present residence, education, economic status, smoking, alcoholics, and total calories intake
Tan et al. (31)	US	40–80	NR	NR	M:411 F:12	M:1,796 F:1,630	FFQ/self-report	NR	1 1.10 (0.88–1.37) 0.88 (0.67–1.16)	Age, gender, race/ethnicity, smoking status, alcohol status, body mass index, *H. pylori* infection, and total energy intake
Toorang et al. (32)	Iran	≥40	64.3 ± 12.2	53.9 ± 11.6	M:158 F:59	M:132 F:55	FFQ/interview	NR Cases: Per 249 mg/d Controls: Per 246 mg/d	1 1.88 (1.09–3.2) 2.22 (1.28, 3.85) Continuous: (1.00, 1.01)	Age, gender, energy, education, smoking, and body mass index
Zhu et al. (14)	China	NR	64.1 ± 10.8	64.0 ± 11.3	M:1,401 F:499	M:4,713 F:1,819	FFQ/interview	<107.24 mg/d 107.24–207.21 mg/d 207.21–352.09 mg/d >352.09 mg/d Per 250 mg/d	1 1.06 (0.87, 1.29) 1.32 (1.08, 1.61) 1.57 (1.26, 1.96) Continuous 1.13 (1.06, 1.22)	Study area, age, gender, education level, income 10 years ago, smoking, alcohol consumption, family history of stomach cancer, H. pylori infection, BMI, exercise 10 years ago, dietary sodium intake, and total energy intake
Harrison et al. (25)	US	NR	62 + 11.7	54.2 + 13.5	M:24 F:67	M:62 F:70	FFQ/self-report	NR	Intestinal: 1 (0.7–1.4) Diffuse: 1 1.3 (0.9–1.8)	Age, gender, calorie intake, race, education, smoking, alcohol drinking, BMI
Wu et al. (33)	US	30–74	NR	NR	M/F:192	M/F:343	FFQ/interview	NR	Gastric cardia: 1 1.73 (0.8–3.9) 1.71 (0.8–3.8) 1.83 (0.8–4.0) Distal gastric 1 2.16 (0.95–4.9) 2.20 (0.98–4.9) 2.90 (1.3–6.3)	Age, sex, race, birthplace, education, smoking, body size, reflux, use of vitamins and total calories

*OR, Odds Ratio; CI, confidence interval; GC, gastric cancer; M, male; F, female; FFQ, food frequency questionnaire; BMI, body mass index; US, United States; NR, not-reported; wk, week*.

* *Presented as mean or rang*.

### Meta-Analysis

**Figure 2:**
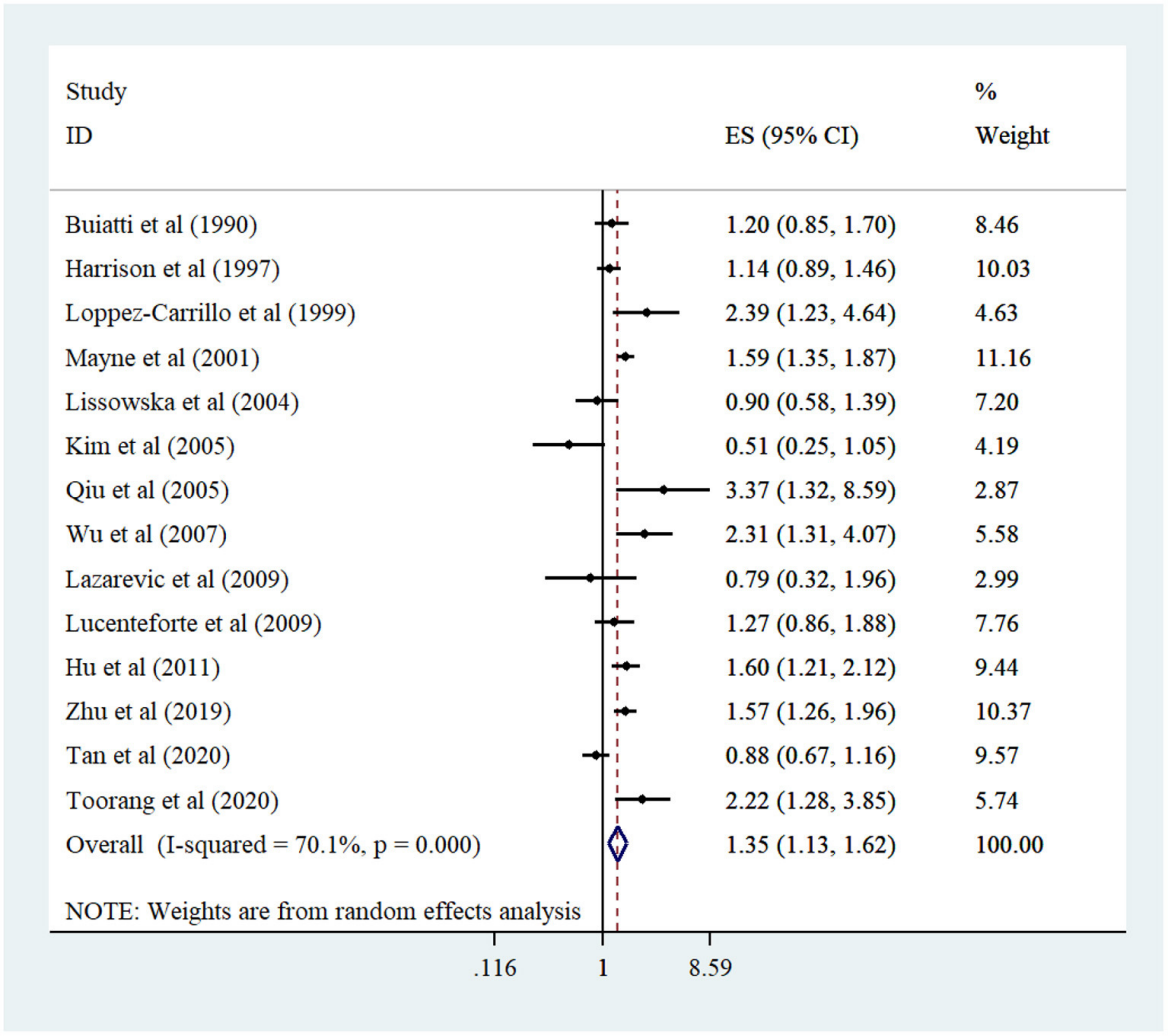
Forest plot derived from random-effects meta-analysis of studies investigating the association between high vs. low intake of dietary cholesterol and gastric cancer in adults. CI, confidence interval; ES, effect size.

In total, 14 case–control studies (13–16, 24–33) were included in the analysis of the highest vs. the lowest dietary cholesterol intake and risk of GC. The meta-analysis indicated an increased risk of GC among participants who consumed the greatest amount of cholesterol compared to participants with the lowest cholesterol intake (pooled OR: 1.35, 95% CI: 1.29–1.62, *I*^2^ = 68%; 95% CI: 45–81%) (Figure 2). Subgroup analysis and meta-regression failed to detect potential sources of heterogeneity. Furthermore, subgroup analysis indicated a positive relationship between dietary cholesterol and GC in population-based case–control studies, studies conducted in non-US countries, those with a higher number of GC patients (≥400), high-quality studies, those that collected dietary data through interviews, studies not adjusted for *H. pylori* infection, and studies where the BMI was controlled (Table 2). In addition, sensitivity analysis did not show evidence for the impact of each study on the overall risk estimate (Supplementary Figure 1). No evidence of publication bias was observed through the Egger test (*P* = 0.83) and funnel plot (Supplementary Figure 2).

**Table 2:** Summary risk estimates for the association between cholesterol intake and risk of gastric cancer in adults aged ≥18 years in case-control studies^a^.

	**#RR^b^**	**Pooled RR (95% CI)^c^**	***I*^**2**^ (%)^d^**	***P*-heterogeneity^e^**	**Meta-regression**
**The highest vs. lowest comparison**					
**Dietary cholesterol intake**					
Overall	14	1.35 (1.129–1.62)	70.1	<0.001	–
Subgroup analysis					
**Study location**					
US	4	1.32 (0.94–1.84)	83.8	<0.001	0.898
Non-US	10	1.37 (1.08–1.74)	63.3	0.004	
**Study design**					
Population-based case-control study	9	1.45 (1.17–1.79)	72	<0.001	0.309
Hospital-based case-control study	5	1.13 (0.78–1.64)	64.5	0.024	
**Study quality**					
High quality	9	1.56 (1.28–1.89)	53.1	0.029	0.146
Low quality	5	1.01 (0.73–1.41)	76.3	0.002	
**Number of cases**					
<400	9	1.37 (1.00–1.89)	69.6	0.001	0.664
≥400	5	1.35 (1.08–1.68)	74.9	0.003	
**Exposure reporting**					
Interview	11	1.43 (1.16–1.77)	64	0.002	0.441
Self-report	3	1.16 (0.84–1.61)	77.5	0.012	
**Adjustment for** ***H. pylori***					
Yes	4	1.17 (0.71–1.91)	85.6	<0.001	0.471
No	10	1.41 (1.17–1.70)	56.9	0.013	
**Adjustment for BMI**					
Yes	8	1.35 (1.14–1.61)	67.6	0.003	0.947
No	6	1.35 (0.77–2.39)	76.9	0.001	
**Dietary cholesterol intake (per 100 mg/d increase)**					
Overall	8	1.05 (0.99–1.12)	83.5	<0.001	
Subgroup analysis					
**Study design**					
Population-based case-control study	5	1.10 (1.04–1.16)	39.7	0.156	0.153
Hospital-based case-control study	3	0.96 (0.83–1.12)	72.8	0.025	
**Study quality**					
High quality	6	1.07 (0.99–1.15)	74.8	0.001	0.222
Low quality	2	0.93 (0.64–1.35)	90.7	0.001	
**Number of cases**					
<400	5	1.01 (0.90–1.13)	71.2	0.008	0.335
≥400	3	1.10 (1.05–1.15)	30	0.24	
**Adjustment for** ***H. pylori***					
Yes	2	0.93 (0.64–1.35)	90.7	0.001	0.407
No	6	1.07 (0.99–1.15)	74.8	0.001	
**Adjustment for BMI**					
Yes	5	1.07 (1.00–1.15)	86.7	<0.001	0.421
No	3	0.97 (0.74–1.26)	83.8	0.002	

a *BMI, body mass index; CI, confidence interval; RR, Relative Risk; FFQ, food frequency questionnaire; US, United States*.

b *Number of risk estimates*.

c *Obtained from the random-effects model*.

d *Inconsistency- the percentage of variation across studies due to heterogeneity*.

e *Obtained from the Q-test*.

**Figure 3:**
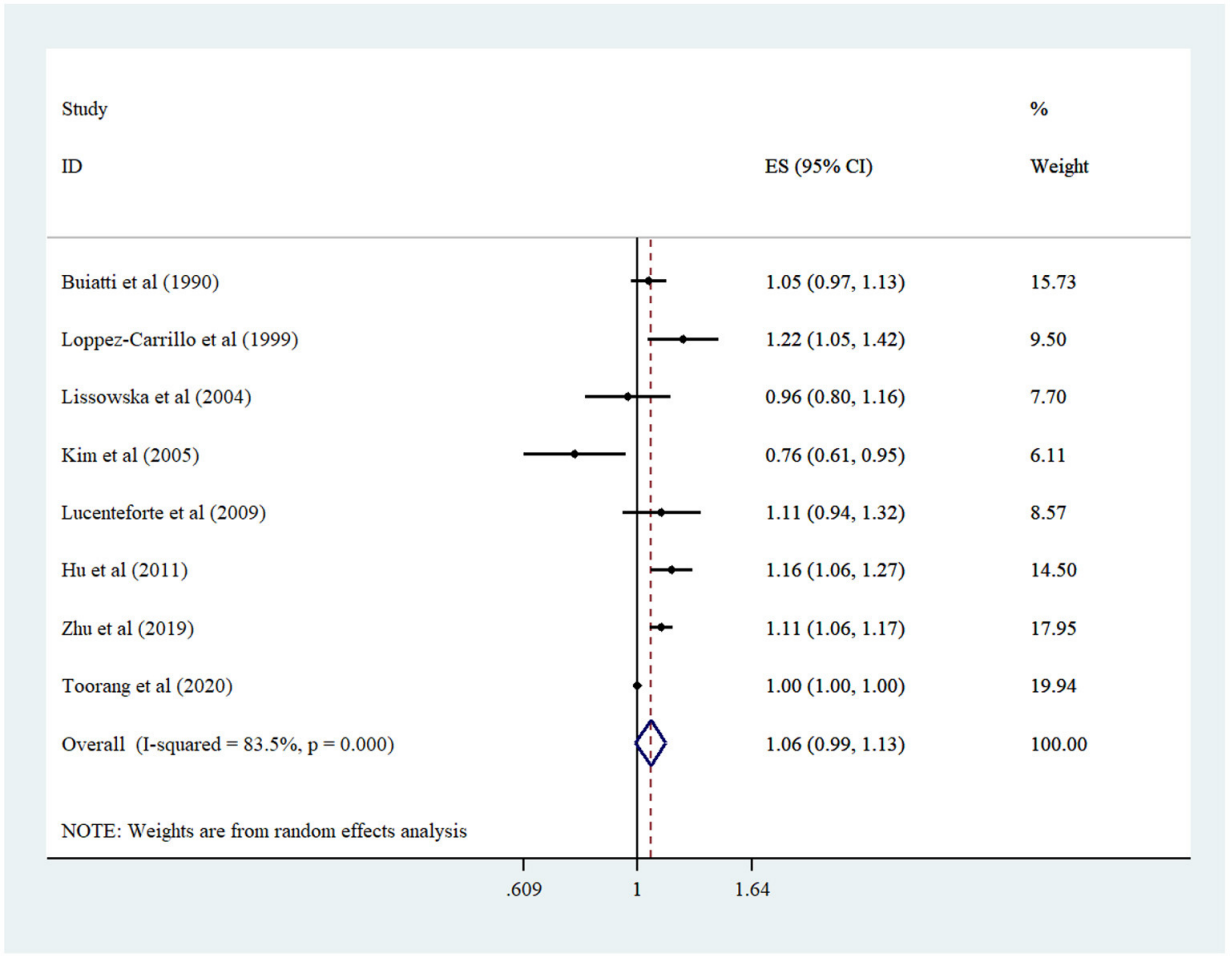
Forest plot derived from random-effects meta-analysis of studies investigating the association between 100 mg/d increment in cholesterol intake and gastric cancer in adults. CI, confidence interval; ES, effect size.

Findings from linear dose–response analysis demonstrated that a 100 mg/d increment in cholesterol intake was not associated with the risk of GC (pooled OR: 1.05, 95% CI: 0.99–1.12, *I*^2^ = 84%; 95% CI: 69–91%) (Figure 3). Sensitivity analysis was done to assess the effect of each study on the overall effect size (Supplementary Figure 3). Because the study of Toorang et al. had a major effect on the main analysis, we repeated the analysis once without it. Here, a marginally significant association was identified between a 100 mg/d increment in cholesterol intake and GC (pooled OR: 1.07, 95% CI: 1.00–1.15, *I*^2^ = 65%; 95% CI: 22–85%). The study design and the number of cases were sources of heterogeneity in the subgroup analysis. Besides, a positive association was seen in population-based case–control studies, studies with higher cases, and studies adjusted for BMI (Table 2). Moreover, there was no evidence of publication bias in the Egger test (*P* = 0.18) and funnel plot (Supplementary Figure 4).

**Figure 4:**
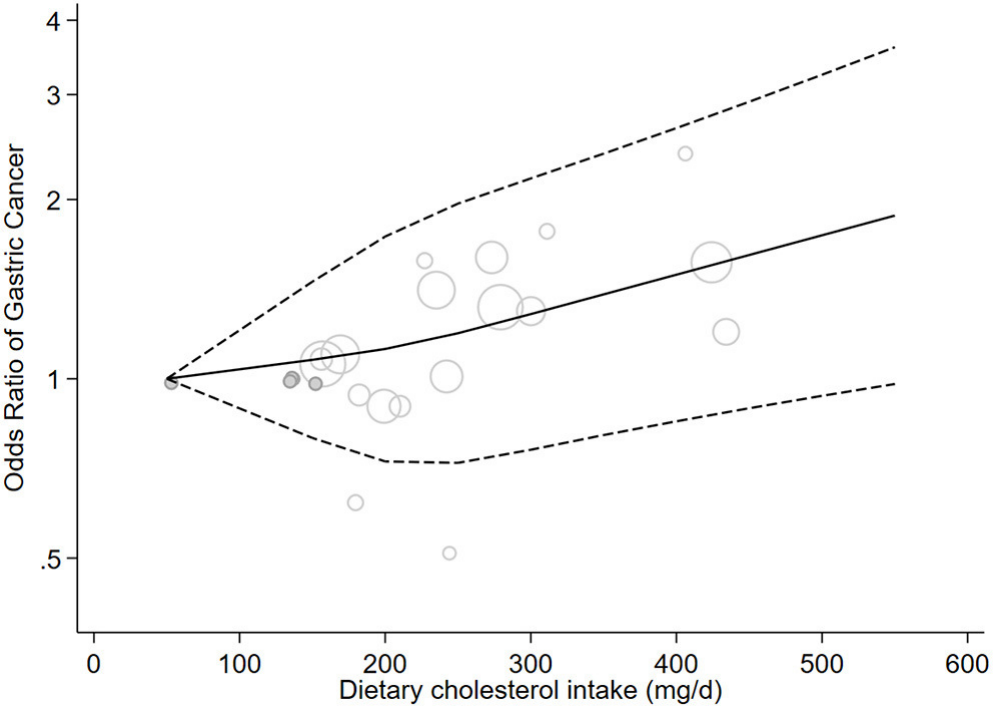
Non-linear dose-response meta-analysis of case-control studies investigating the association between cholesterol consumption and risk of gastric cancer in adults (*P* = 0.03).

A non-linear dose–response association was observed between dietary cholesterol intake and the risk of GC (*P* = 0.03; Figure 4).

## Discussion

In this systematic review and meta-analysis of 14 case–control studies, we found that higher intakes of dietary cholesterol were associated with a 35% greater risk of GC among adults. In addition, a non-linear dose–response relationship was observed. This study is the first systematic review and meta-analysis to examine the relationship between cholesterol intake and the risk of GC.

Cholesterol plays a vital role in maintaining cellular homeostasis in the body (34). Major dietary sources of cholesterol include red meat, processed meat, egg yolks, dairies, fish, butter, cheese, shrimp, and poultry (35). Considering that a high-cholesterol diet might represent an unhealthy dietary pattern and lead to chronic diseases such as cancer and cardiovascular diseases (36, 37), the relationship between dietary cholesterol and the risk of cancer has received much attention (11, 12). This meta-analysis suggests that high dietary cholesterol intake may elevate the odds of GC. In line with our finding, one hospital-based case–control study in Spain found a positive relationship between cholesterol consumption and GC (38). Jung et al. (39) also expressed that high serum cholesterol was linked to the incidence of GC. Furthermore, some meta-analyses found a significant positive association between dietary cholesterol intake and cancers of the ovaries, breasts, pancreas, esophagus, and lungs (9–12, 34).

In contrast, in two meta-analyses, intake of red meat and eggs (rich sources of cholesterol) was not associated with the risk of GC (40, 41). Given that cholesterol is consumed in combination with other compounds such as salt, nitrates, multivitamins, minerals, and high-quality protein, the interaction between different nutrients prevents us from understanding the individual effect of cholesterol. We know that cholesterol is found in animal foods and high-cholesterol diets are poor sources of plant foods, including fruits and vegetables. Evidence indicates that people who consume high amounts of vegetables and fruits have a lower risk of GC (42, 43). This effect might be due to the presence of many antioxidants (particularly vitamin C, vitamin E, and carotenoids) in fruits and vegetables, which possess anticarcinogenic properties (44). In addition, an inverse association was seen between serum cholesterol concentrations and the occurrence of GC in some cohort studies (45, 46). The amount of cholesterol in cancer cells is higher than the normal cells, and cholesterol helps in cancer promotion (47). It is still ambiguous whether low serum cholesterol is a cause or effect in relation to GC, and this issue needs to be examined. Therefore, it is likely that dietary cholesterol increases the risk of cancer without augmenting blood cholesterol levels.

The inconsistencies among studies may be explained by variations in study design, geographic regions, adjustments, reporting of dietary data, quality of studies, and/or the number of cases. It has been shown that *H. pylori* infection, smoking, alcohol consumption, obesity, salt-rich diet, nitrites, and hot meals are the determinants of GC (48, 49). High dietary cholesterol intake may take part in GC initiation or progression by supporting *H. pylori* infection. *H. pylori* infection leads to gastric atrophy and hypochlorhydria, which promote the colonization of acid-intolerant bacteria (50) and elevate the occurrence of GC (51). Our findings indicated no association between dietary cholesterol intake and GC after adjusting our results for *H. pylori* infection. Furthermore, most of the included studies were adjusted for smoking and energy intake, which are the critical risk factors of GC. Besides, we found a significant positive association between cholesterol intake and GC in studies adjusted for BMI.

There are some potential mechanisms regarding the relationship between cholesterol and GC. Dietary cholesterol might play a role in cancer development via changes in lipid metabolism, which are related to cellular inflammation (52). An increase in total cholesterol and LDL as well as a decrease in HDL could induce the production of inflammatory biomarkers such as interleukin-6 and tumor necrosis factor-α (53).

This study possessed some strengths. First, linear and non-linear dose–response analyses help us to reveal the shape and strength of probable association. Second, most of included studies applied an interview-administered questionnaire. Self-reported questionnaires for cholesterol intake assessment might inevitably lead to some misclassification of participants in terms of exposure. Third, most studies took into account a wide range of important confounding factors, including energy intake, smoking, alcohol consumption, and BMI. Finally, publication bias was not detected. Nonetheless, our study had some limitations. First, based on our knowledge, there was no cohort study to examine the association between dietary cholesterol and GC. Because case–control studies have diverse kinds of bias, including selection bias, recall bias, and measurement bias, the case–control nature of included studies prevented us from reaching a decisive conclusion. Second, some fundamental residual confounders such as *H. pylori* infection, dietary factors (salt, nitrates, etc.), and lipid-lowering medications (especially statin use) were ignored in the adjustments of most studies. Third, although we tried to detect the sources of heterogeneity among studies, we could not find them through subgroup analysis and meta-regression. Due to a limited number of studies, we could not perform subgroup analysis for other potential relevant factors. Finally, measurement errors are unavoidable in estimates of dietary cholesterol intake.

In conclusion, this review illustrated an association between high dietary cholesterol intake and GC development in case–control studies. This study suggests the importance of dietary cholesterol modification in the prevention of GC. Considering that all of the included studies had case–control designs prone to biases, these results warrant cohort investigations. Large, long-duration, prospective cohort studies that consider the important dietary and non-dietary covariates are obligatory to achieve a comprehensive understanding of this matter.

## Author Contributions

PM and LG designed the work, extracted the data, analyzed the data, and critically reviewed the manuscript. LG wrote the first draft of the manuscript. Both authors contributed to the article and approved the submitted version.

## Conflict of Interest

The authors declare that the research was conducted in the absence of any commercial or financial relationships that could be construed as a potential conflict of interest.

## Publisher's Note

All claims expressed in this article are solely those of the authors and do not necessarily represent those of their affiliated organizations, or those of the publisher, the editors and the reviewers. Any product that may be evaluated in this article, or claim that may be made by its manufacturer, is not guaranteed or endorsed by the publisher.
