# Supplementary material for: Association of Dietary Cholesterol Intake With Risk of Gastric Cancer: A Systematic Review and Meta-Analysis of Observational Studies
Source: Front Nutr. 2021 Aug 12;8:722450. doi: 10.3389/fnut.2021.722450 (PMC8387575; doi:10.3389/fnut.2021.722450)
Supplement: Supplementary file 1 [file Data_Sheet_1.docx]

**Supplementary Figure 1**

Sensitivity analysis of case-control studies assessing the association between cholesterol consumption and risk of gastric cancer in adults. CI, confidence interval

**Supplementary Figure 2**


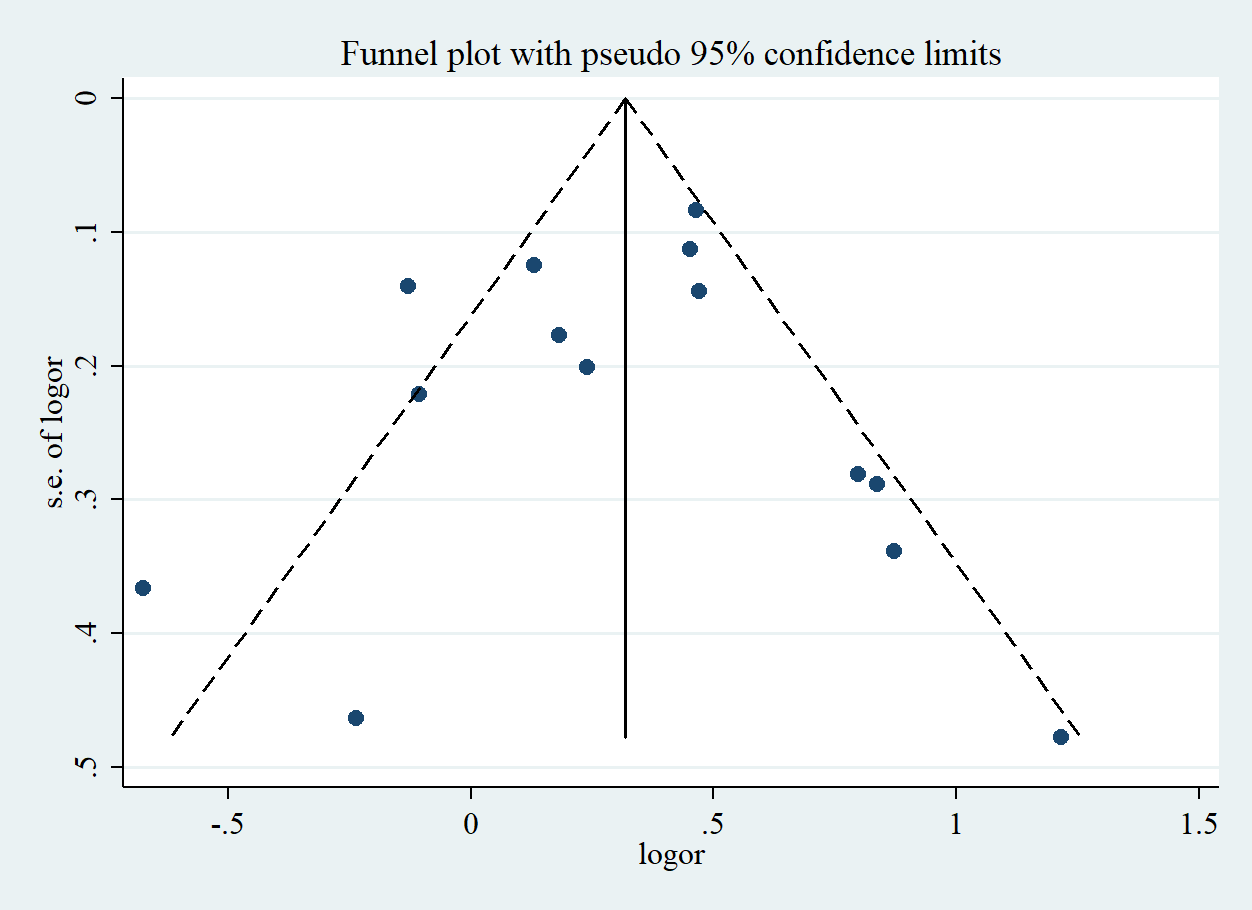


Funnel plot of the risk estimates of fourteen studies on the association between cholesterol consumption and risk of gastric cancer in adults. Each dot represents one study. Egger’s test P=0.83. Log RR: natural logarithm of relative risk. CI, confidence interval; SE, standard error.

**Supplementary Figure 3**

Sensitivity analysis of case-control studies assessing the association between 100 mg/d increment in cholesterol consumption and risk of gastric cancer in adults. CI, confidence interval

**Supplementary Figure 4**


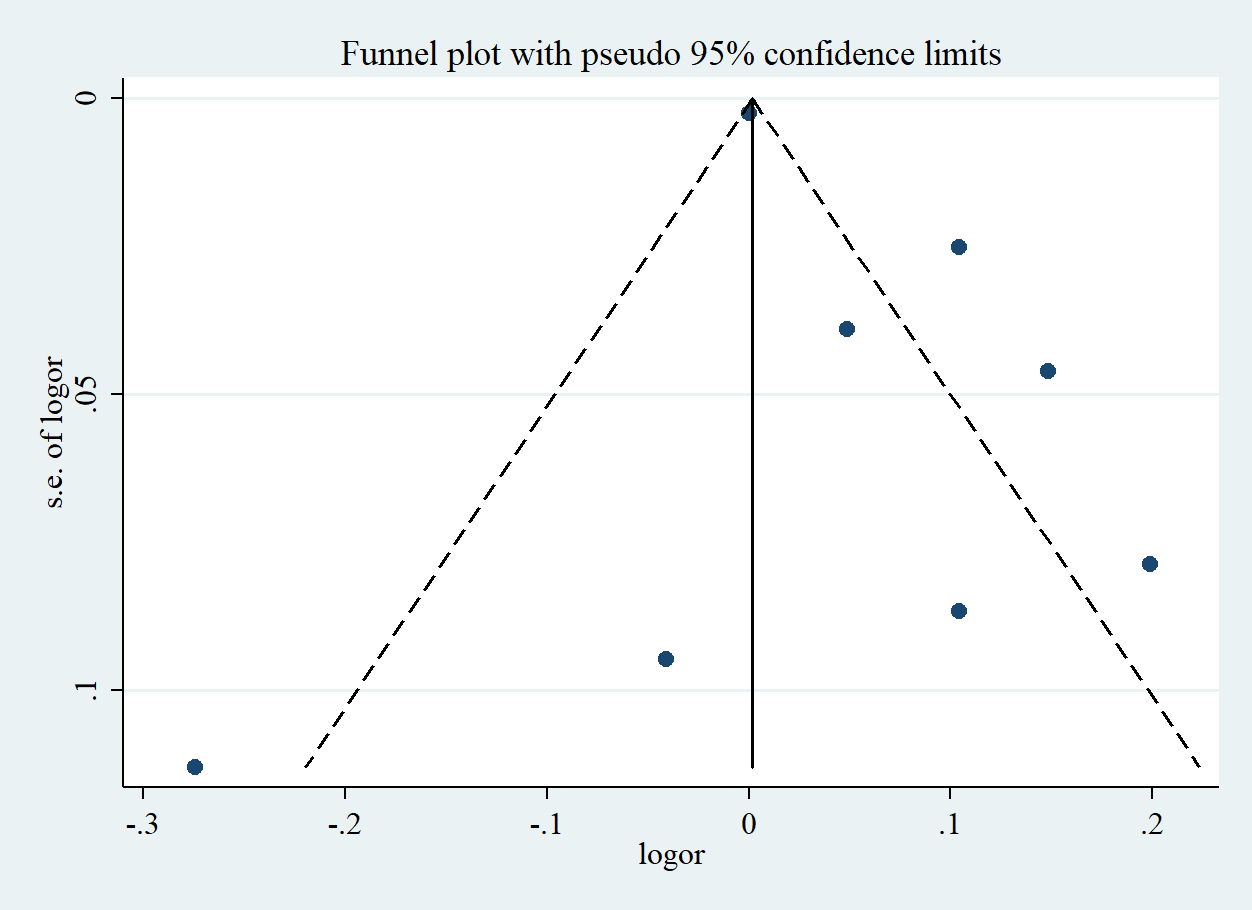


Funnel plot of the risk estimates of eight studies on the association between 100 mg/d increment in cholesterol consumption and risk of gastric cancer in adults. Each dot represents one study. Egger’s test P=0.18. Log RR: natural logarithm of relative risk. CI, confidence interval; SE, standard error.
